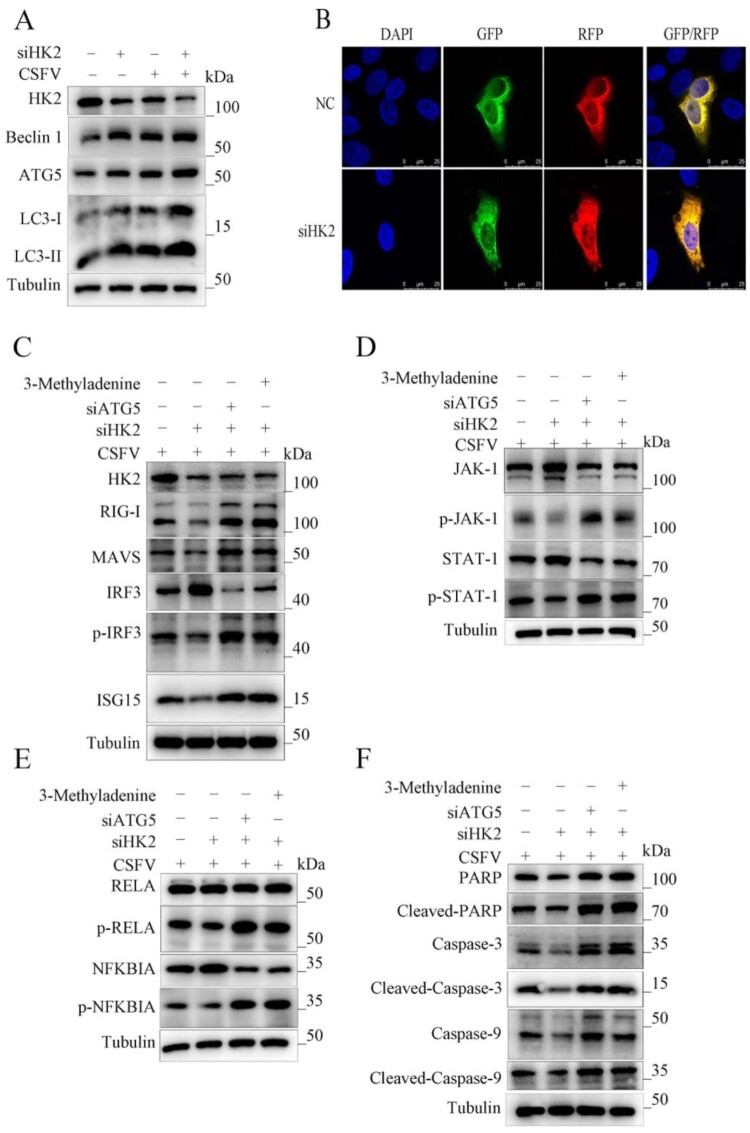# Correction

**DOI:** 10.1080/22221751.2024.2445896

**Published:** 2025-01-06

**Article title:** The regulation of cell homeostasis and antiviral innate immunity by

autophagy during classical swine fever virus infection

**Authors:** Li, X., Song, Y., Wang, X., Fu, C., Zhao, F., Zou, L., Wu, K., Chen, W., Li, Z., Fan, J., Li, Y., Li,

B., Zeng, S., Liu, X., Zhao, M., Yi, L., Chen, J., and Fan, S.

**Journal:** *Emerging Microbes & Infections*

Volume 12 Issue 1

**DOI:** https://doi.org/10.1080/22221751.2022.2164217

When this article was first published online, figures 4, 6, 7, and 8 contained errors. These figures have now been corrected in the online version.

Wrong figures:

**Figure 4.**
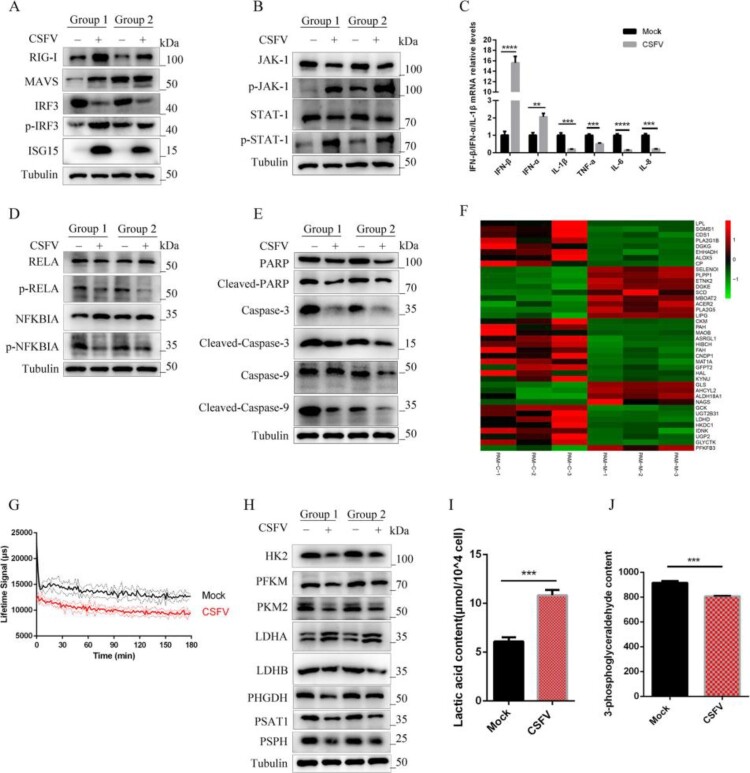

**Figure 6.**
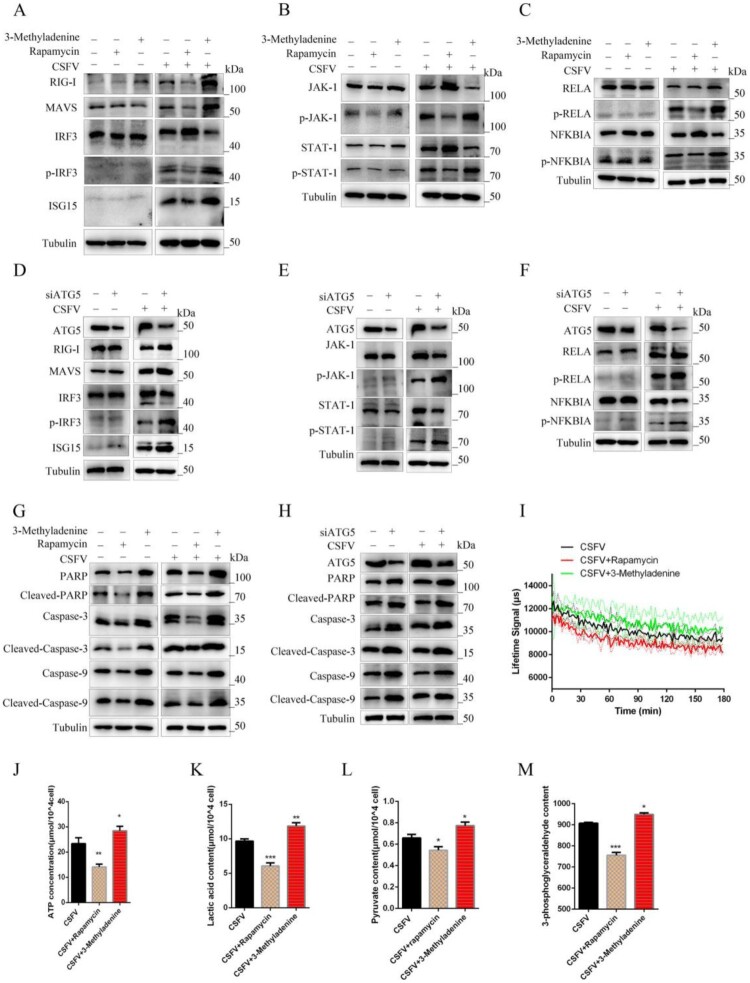

**Figure 7.**
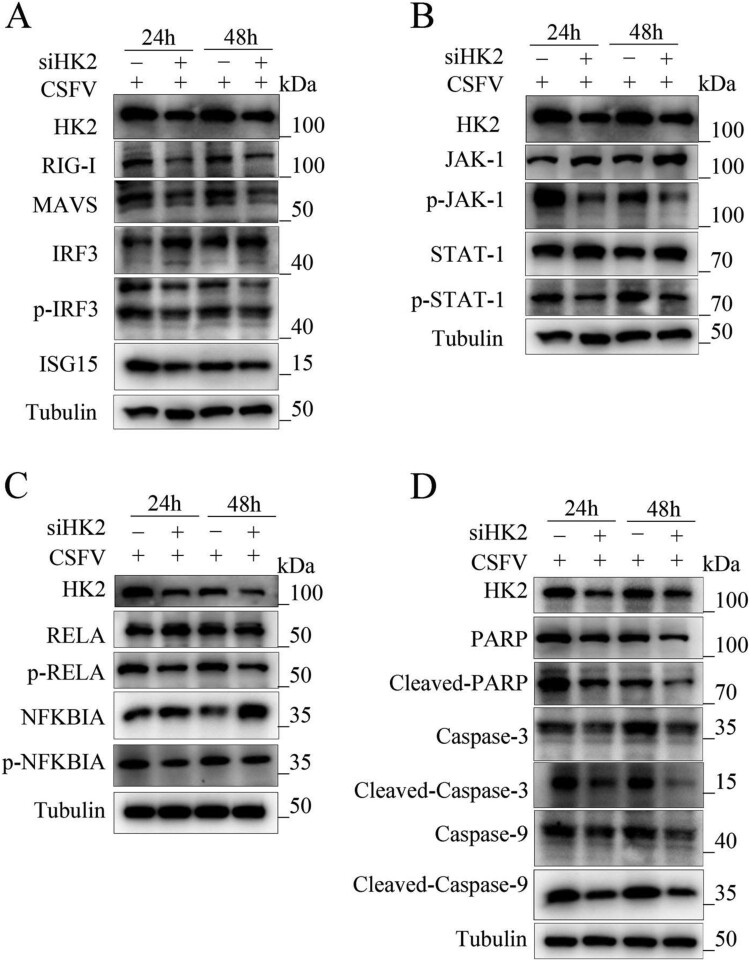

**Figure 8.**
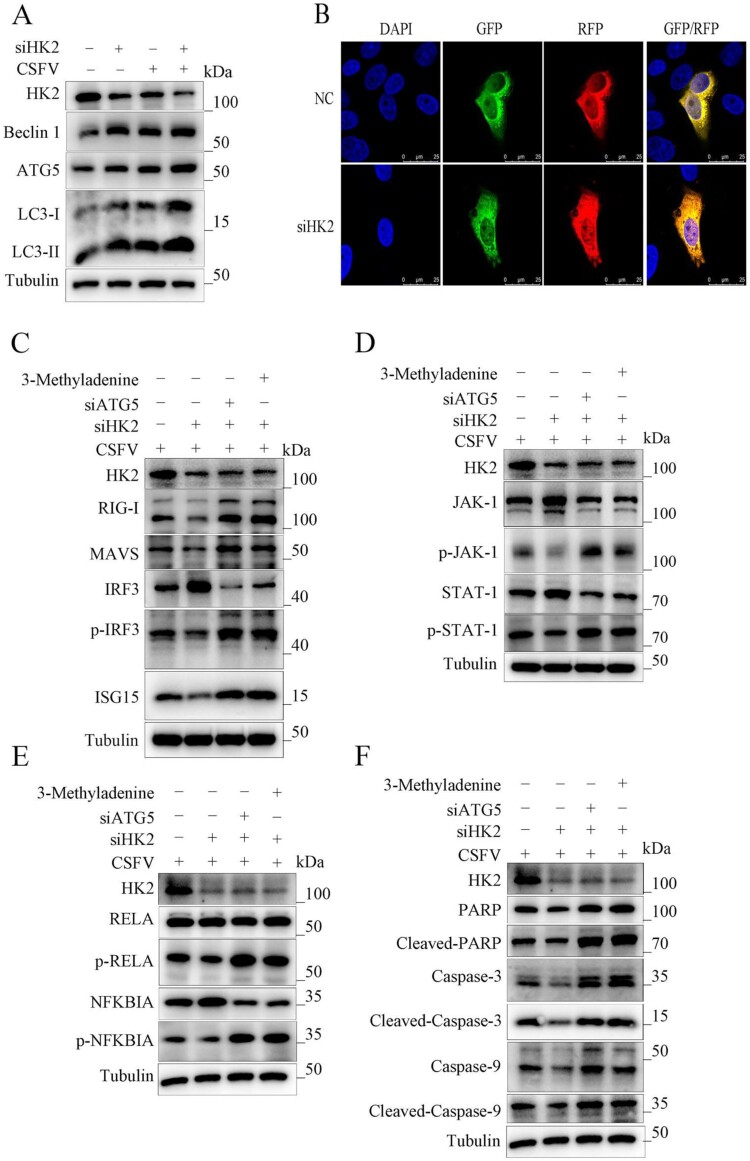

Correct Figures:

**Figure 4.**
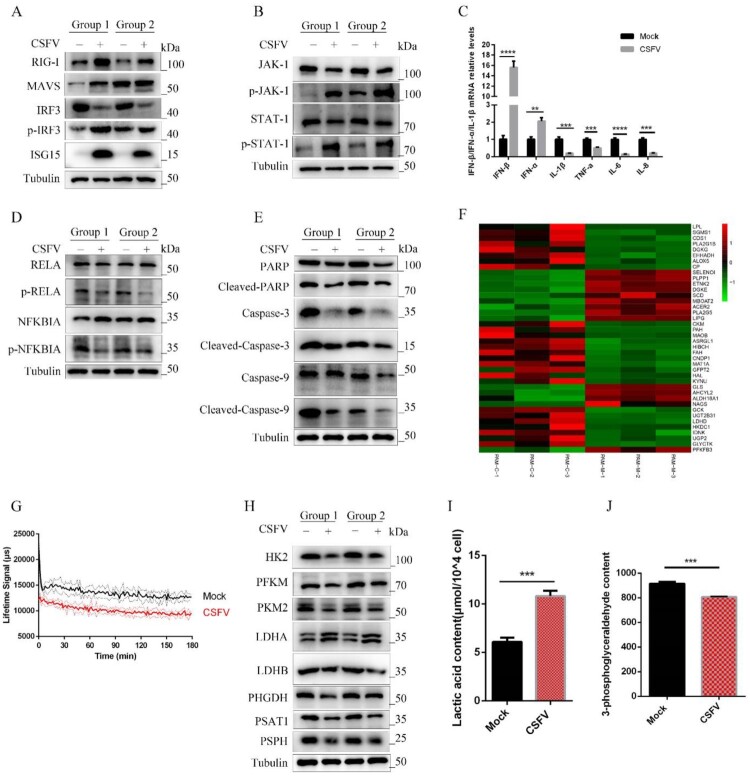

**Figure 6.**
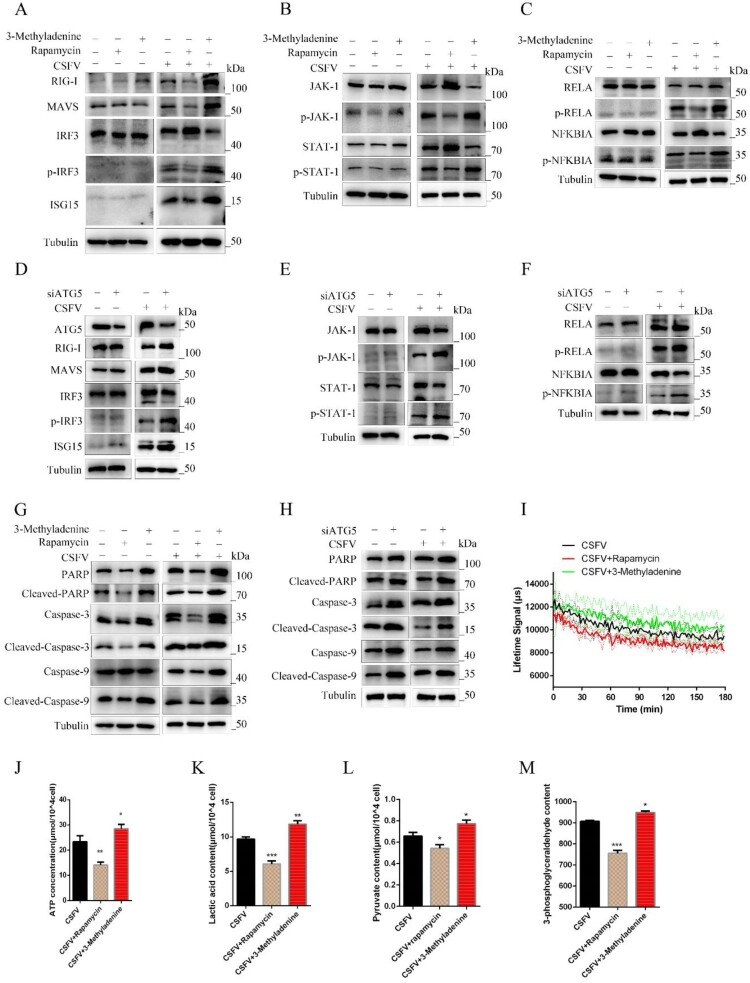

**Figure 7.**
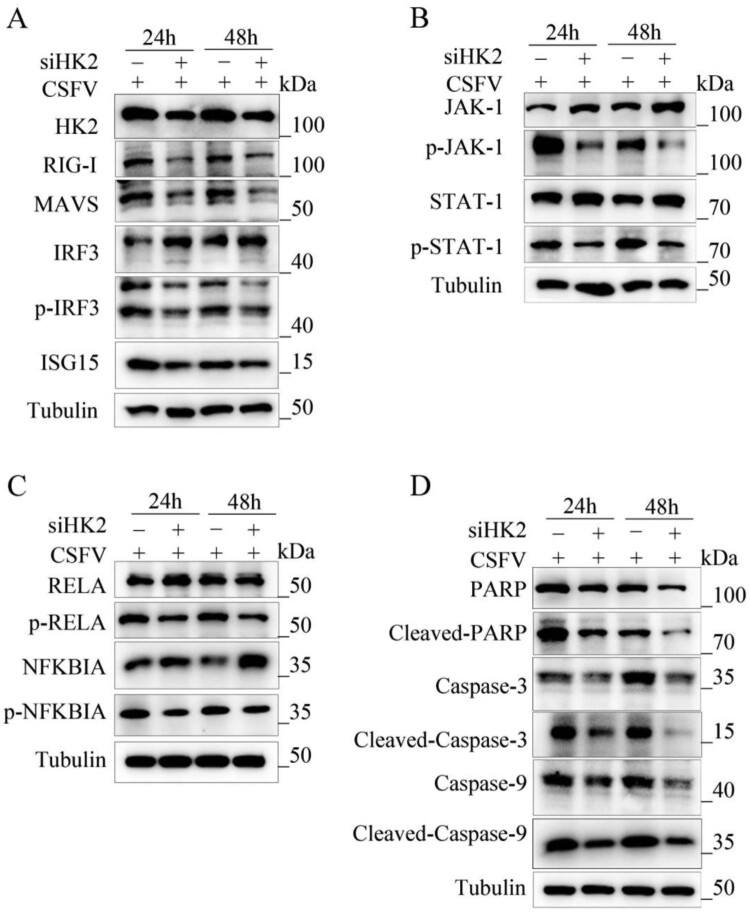

**Figure 8.**